# Geographic and outcome variation among black men diagnosed with prostate cancer

**DOI:** 10.1186/1750-9378-6-S2-S2

**Published:** 2011-09-23

**Authors:** Camille Ragin, Batsirai Mutetwa, Alison Attong-Rogers, Veronica Roach, Emanuela Taioli

**Affiliations:** 1 Cancer Prevention and Control Program, Fox Chase Cancer Center, Philadelphia, Pennsylvania, USA; 2 SUNY Downstate Medical Center, Brooklyn, NY, USA; 3 SUNY Downstate Medical Center Cancer Registry, University Hospital of Brooklyn, New York, USA; 4 Dr Elizabeth Quamina National Cancer Registry of Trinidad and Tobago, Mt. Hope, Trinidad; 5 Institute of Translational Epidemiology, Mt. Sinai Medical Center, New York 10029, USA

## Abstract

**Background:**

Prostate cancer is the sixth leading cause of death from cancer among men worldwide. We have previously reported that prostate cancer survival rates for Caribbean-born males in the US was similar to survival rates of African-Americans and was higher than their counterparts diagnosed in the Caribbean. However, it is not clear whether differences in mortality could be attributed to differences in treatment.

**Methods:**

This current analysis seeks to further explore reasons for the geographic variation of prostate cancer survival for Caribbean-born men. This analysis included 2,554 Black newly diagnosed prostate cancer cases (960 cases diagnosed in the US and 1,594 cases diagnosed in the Caribbean). Clinical data were extracted from the cancer registry and clinical charts.

**Results:**

There were noted differences in the pattern of treatment for each place of birth category when stratified according to disease stage at diagnosis. Among the patients diagnosed with early-intermediate disease (stage I-III) the majority of US-born Brooklyn men were treated with surgery only (31%) and a similar pattern was observed for Caribbean-born Brooklyn men (35%). In contrast, the majority of Caribbean-born Trinidad & Tobago men were treated with hormone therapy (31%).

Caribbean-born men diagnosed in the Caribbean had a significantly higher risk of death from prostate cancer (Adjusted Hazard [AdjHR]: 3.7, 95% Confidence Interval [CI]: 2.8-5.0) when compared with the risk of death for Caribbean-born males diagnosed in the US. This observation was consistent for each treatment group with the exception of the cases treated with hormone therapy only. For these cases, there was no difference in the risk of death between Caribbean-born males diagnosed in the Caribbean (AdjHR: 1.4, 95% CI: 0.8-2.6) compared to Caribbean-born males diagnosed in the US.

**Conclusions:**

In addition to difference in access and utilization of screening, differences in treatment strategy may also be a strong predictor of outcome for Caribbean-born males diagnosed with prostate cancer. Further studies are needed to confirm these findings. In addition, other environmental factors related to survival that was not considered in this analysis also need to be investigated.

## Background

Prostate cancer is the sixth leading cause of death from cancer among men worldwide [1]. The age-adjusted death rates are highest in black populations within the United States (US) (52.0 per 100,000 in 2007) [2], and in other geographic regions such as the Caribbean (26.3 per 100,000 in 2008) and Sub-Saharan Africa (18.5 per 100,000 in 2008) [1]. In a previously published study, cancer registry data from the US (Brooklyn) and the Caribbean (Trinidad & Tobago and Guyana) were evaluated to determine the contribution of place of birth to prostate cancer survival rates among black men [3]. We reported after adjusting for age and stage at diagnosis that survival rates were lower for Black men diagnosed in the Caribbean compared to black men diagnosed in the US and that Caribbean-born males diagnosed in the US had similar survival rates as African-Americans. It is possible that the difference in outcome between these two groups of Caribbean-born men may be explained by higher utilization of screening and/or easier access to care and to efficacious treatment in the US. This current analysis seeks to further explore the geographic variation of prostate cancer survival for Caribbean men in order to shed some insight on the previous findings and to confirm this hypothesis. Further explorations were made to determine whether the geographic variation of prostate cancer survival for Caribbean-born men may be attributed to differences in treatment strategy.

## Methods

De-identified cancer registry datasets were obtained for all men diagnosed with histologically confirmed prostate cancer between 1995 and 2007 from the US (all cases diagnosed and/or treated at the University Hospital of Brooklyn, Brooklyn, New York, USA) and Caribbean (all cases diagnosed and treated in Trinidad & Tobago). Detailed methods related to data collection and coding were previously described [3]. The cancer registry study population (N = 5,533) consisted of 1,100 males diagnosed and/or treated in the US and 4,433 males diagnosed and treated in the Caribbean (Trinidad & Tobago). This current analysis was limited to Black males only (N = 3,424). Among these, 662 cases (19%) diagnosed in the Caribbean and 392 cases (11%) diagnosed in the US were excluded because place of birth was not specified and 208 cases (6%) diagnosed in the Caribbean were excluded because treatment data were not available. Therefore the final number of cases included in this analysis was 2,162 prostate cancer cases (568 cases diagnosed in the US and 1,594 cases diagnosed in the Caribbean).

Statistical analyses were performed using STATA SE (version 10.1) software (StataCorp. LP, College Station, TX, USA). Place of birth for all cases was classified as US-born Brooklyn, Caribbean-born Brooklyn, African-born Brooklyn and Caribbean-Born Trinidad & Tobago. Marital status was categorized as single, married (including common-law marriage) and other (which includes separated, divorced and widowed). For comparison between cancer registry datasets, stage at diagnosis was reclassified as Stage I-III (localized or regional) and Stage IV (distant). Treatment was categorized as having no treatment (i.e. no surgery, radiotherapy or hormone therapy), surgery only, radiotherapy only, hormone therapy only or combination therapy (surgery and/or radiotherapy and/or hormone therapy). Follow up time was calculated in months by subtracting the date of diagnosis from the date of last contact (for cases that were alive) or from the date of expiration (for cases that were dead). All statistical tests were two-sided, and a P-value less than 0.05 were considered statistically significant.

One-way analysis of variance was performed in order to compare the differences in mean age across categories of place of birth. Associations between place of birth and stage at diagnosis or treatment were analyzed using the chi-square test. Multivariable cox proportional hazard models estimated the risk of death for each place of birth category according to the type of treatment received.

## Results

### Stage at diagnosis and treatment according to place of birth

The majority of the cases were married and there were no significant differences according to place of birth. The mean age at diagnosis was similar for men diagnosed in the US irrespective of their place of birth (US-born Brooklyn men: 65.1 years, standard deviation [SD] ± 9.7; Caribbean-born Brooklyn men: 66.2 years, SD ± 8.5 and African-born Brooklyn men: 64.6 ± 9.8) while the mean age at diagnosis for Caribbean-born men in Trinidad & Tobago were significantly higher than men diagnosed in the US (70.4 years, SD ± 9.5) (test of equal variance, p – value: 0.044). For both the US and Trinidad & Tobago males, adenocarcinoma was the most common histologic type that was diagnosed. The majority of cases were diagnosed at stage I-III irrespectively of their place of birth (Table 1). However, twenty-one percent of Caribbean-born males diagnosed in Trinidad and Tobago had advanced disease (stage IV) while the proportion of stage IV disease among males diagnosed in Brooklyn varied from 7% for Caribbean born men living in the US to 10% for US born men **.** Further stratification of the cases diagnosed in Trinidad & Tobago revealed that the proportion of early-intermediate stage and late-stage cancers were different between the two islands. For Tobago, 75% of the cases were diagnosed with early-intermediate stage disease and 11% diagnosed with late stage disease, 14% of the cases were not staged. In contrast, 56% of the cases in Trinidad were diagnosed with early-intermediate disease, 24% were diagnosed with late stage disease and 20% were not staged.

**Table 1:** Clinical and treatment characteristics of prostate cancer cases

Characteristic	US-born Brooklyn N = 125 N (%)	African-born Brooklyn N = 37 N (%)	Caribbean-born Brooklyn N = 406 N (%)	Caribbean-born Trinidad &Tobago N = 1,594 N (%)	P-Value
*Histology*					
Carcinoma, NOS	4 (3.2)	2 (5.4)	5 (1.2)	177 (11.1)	<0.0001
Adenocarcinoma	121 (96.8)	35 (94.6)	397 (97.8)	1,406 (88.2)	
Other^1^	0 (0.0)	0 (0.0)	4 (0.99)	11 (0.7)	
*Stage*					
I-III	111 (88.8)	32 (86.5)	367 (90.4)	953 (59.8)	<0.0001
IV	12 (9.6)	3 (8.1)	27 (6.7)	338 (21.2)	
Unstaged	2 (1.6)	2 (5.4)	12 (3.0)	303(19.0)	
*Treatment*					
*Stage I-III*	N = 111	N = 32	N = 367	N = 953	<0.0001
No treatment	32 (28.8)	9 (28.1)	54 (14.7)	250 (26.2)	
Surgery only	34 (30.6)	10 (31.2)	128 (34.9)	107 (11.2)	
Radiotherapy only	29 (26.1)	5 (15.6)	83 (22.6)	64 (6.7)	
Hormone therapy only	0 (0.0)	0 (0.0)	13 (3.5)	295 (31.0)	
Combination therapy*	16 (14.4)	8 (25.0)	89 (24.3)	237 (24.9)	
*Treatment*					
*Stage IV*	N = 12	N = 3	N = 27	N = 338	0.295
No treatment	6 (50.0)	1 (33.3)	9 (33.3)	124 (36.7)	
Surgery only	2 (16.7)	0 (0.0)	1 (3.7)	13 (3.9)	
Radiotherapy only	2 (16.7)	1 (33.3)	2 (7.4)	32 (9.5)	
Hormone therapy only	2 (16.7)	1 (33.3)	9 (33.3)	74 (21.9)	
Combination therapy*	0 (0.0)	0 (0.0)	6 (22.2)	95 (28.1)	

^*^Combination refers to any combination of surgery, radiation and hormone therapy or at least two different types of therapy. ^1^Other (histology): Glandular intraepithelial neoplasia, grade III; Small cell carcinoma; Squamous cell carcinoma; Transitional cell carcinoma; Cribriform carcinoma; Acinar cell carcinoma and Sarcoma.

Among the patients diagnosed with early-intermediate disease (stage I-III) the majority of US-born Brooklyn men were treated with surgery only (31%) and a similar pattern was observed for Caribbean-born Brooklyn men (35%) as well as for Africa-born Brooklyn men (31%). In contrast, the majority of Caribbean-born Trinidad & Tobago men were treated with hormone therapy (31%).

For patients diagnosed with advanced disease (Stage IV) the majority of US-born Brooklyn men had no treatment (50%). In contrast to US-born men diagnosed in Brooklyn, the pattern of treatment appeared to be similar for Caribbean-born Brooklyn men and Caribbean-born Trinidad & Tobago men. A fairly close to equal proportion of patients receiving either no treatment, hormone therapy only or combination therapy was observed for these groups.

### Survival rates and place of birth according to stage at diagnosis and treatment received

Overall survival rates for each place of birth category stratified according to stage at diagnosis revealed differences in outcome for Caribbean men. For cases diagnosed with early/intermediate stage cancer, the five-year survival rates for men diagnosed in Brooklyn were about two-fold higher than that of Caribbean-born men diagnosed in Trinidad & Tobago men, this was irrespective of whether the Brooklyn men were born in the US, Caribbean or Africa (84%, 82% and 77% vs. 41%). In contrast, for the cases diagnosed with advanced disease the 5-year survival rate for US-born men diagnosed in Brooklyn was 49%, while much lower but similar five-year survival rates were seen for Caribbean-born men diagnosed in Brooklyn and Caribbean-born men diagnosed in Trinidad & Tobago (29% and 10% respectively).

Table 2 summarizes the hazard ratios for each place of birth category according to the type of treatment after adjusting for stage and age at diagnosis. For the overall study population, and as previously reported [3], Caribbean-born men diagnosed in the Caribbean had a significantly higher risk of death from prostate cancer (Adjusted Hazard [AdjHR]: 3.8, 95% Confidence Interval [CI]: 2.8-5.1) when compared with the risk of death for Caribbean-born males diagnosed in the US. This observation was consistent for each treatment group with the exception of the cases treated with hormone therapy only. For these cases, there was no difference in the risk of death between Caribbean-born males diagnosed in the Caribbean (AdjHR: 1.4, 95% CI: 0.8-2.6) compared to Caribbean-born males diagnosed in the US. In contrast, the African-born men treated with hormone therapy only were shown to have a significantly increased risk of death compared to the Caribbean-born Brooklyn men. For all other treatment groups, there was no difference in the risk of death between African-born and Caribbean-born men diagnosed in the US. It was not possible to determine the risk of death for US-born males diagnosed in the US because only two cases treated with hormone therapy only. Similarly, the risk of death for African-born males diagnosed in the US and treated with combination therapy could not be estimated because there were too few cases.

**Table 2:** Risk of death among Black men according to type of treatment received

	No treatment N = 493	Surgery only N = 310	Radiotherapy only N = 236	Hormone therapy only N = 449	Combination therapy N = 630
***Place of Birth/registry***	AdjHR (95% CI)^**^	AdjHR (95% CI)^**^	AdjHR (95% CI)^**^	AdjHR (95% CI)^**^	AdjHR (95% CI)^**^
**Caribbean-born Brooklyn**	Reference	Reference	Reference	Reference	Reference
**US-born Brooklyn**	0.9 (0.3-2.9)	0.6 (0.1-3.2)	1.4 (0.6-3.4)	--	1.1 (0.3-4.0)
**Caribbean-born Trinidad & Tobago**	4.4 (2.3-8.5)	3.9 (1.7-9.1)	3.0 (1.3-7.1)	1.4 (0.8-2.6)	5.2 (2.9-9.3)
**African-born Brooklyn**	2.2 (0.5 –10.1)	2.8 (0.3-23.7)	3.3 (0.7-15.0)	6.2 (1.4-28.4)	--

AdjHR = Hazard ratio; *After adjusting for treatment and age at diagnosis and stage –

**adjusting for age and stage

## Discussion

Geographic and outcome variation of prostate cancer survival has been well documented [4]. In this analysis, our aim was to further explore the geographic and outcome variation for prostate cancer for black Caribbean males. Using cancer registry data, we have evaluated whether differences in treatment may account for the observed difference on outcome for Caribbean-born males diagnosed in the US compared to those diagnosed in the Caribbean. In this as well as a previous study, we have shown that Caribbean-born males diagnosed in the US have survival benefit over Caribbean-born males diagnosed in the Caribbean [3]. Possible explanations for this finding may be related to improved access to screening and medical care. We anticipate that a population with adequate access and utilization of screening would subsequently have a higher proportion of cases diagnosed early. This analysis highlights that the majority of cases in the US were diagnosed at stage I-III while Caribbean-born cases diagnosed in the Caribbean were diagnosed mostly with advance stage disease thus supporting the notion that access to screening is likely to contribute to the geographic variation in survival for Black Caribbean-born men. Further stratification of the cases diagnosed in Trinidad & Tobago revealed a much smaller proportion of late stage cancers diagnosed in Tobago (11%), which was similar to that of US men (range according to place of birth, 7-10%) compared to 24% of men diagnosed with late stage cancers in Trinidad. For more than a decade, a longitudinal prostate cancer screening study has been conducted in Tobago and a high prevalence of screening-detected prostate cancer cases has been reported [5]. This may help to explain why there is a higher proportion of early-intermediate stage disease diagnosed in Tobago compared to Trinidad and demonstrates the impact of screening on prostate cancer stage at diagnosis.

In addition to access and utilization of screening, differences in treatment strategy may also be a strong predictor of outcome for Caribbean-born males diagnosed with prostate cancer. Treatment patterns between cases diagnosed with early-intermediate and advanced disease were different. Furthermore, the pattern of treatment correlated with outcome. For early-intermediate stage disease, Caribbean-born males diagnosed in the US had similar treatment patterns as US-born males and both groups showed similar 5-year survival rates (84% vs 82%). In contrast, for cases with late-stage disease, Caribbean-born males diagnosed in the US had dissimilar treatment patterns as US-born males and were more closely related to their counterparts diagnosed in the Caribbean. Both Caribbean-born groups had similar 5-year survival rates.

In general the Caribbean islands are considered developing nations with limited resources; therefore it would not be unusual to assume that there may be limited resources available for adequate cancer care. Our analysis includes data for cases diagnosed in the Caribbean nation of Trinidad & Tobago which unlike most Caribbean islands, has a high-income economy according to the World Bank classification [6] and PSA and DRE screening is available through a public universal health care system provided to the general public. Therefore, it is possible that there may not be as large a disparity in the available resources for cancer treatment compared to the US as it may be for other Caribbean nations. It is important to note that when the cases were stratified according to treatment received, for those cases treated with hormone therapy, there was no difference in outcome between Caribbean-born males diagnosed in the US compared to Trinidad & Tobago. For Caribbean-born males treated in the US, hormone therapy appears to be the more common treatment approach only for advanced disease which is in contrast to their Caribbean counterparts since hormone therapy is more commonly used irrespective of cancer stage. Our findings suggest that treatment might be a strong predictor of outcome for Caribbean-born males. Further studies of the geographic variation of prostate cancer outcome for Caribbean-born men are needed and should also include treatment strategies and other environmental factors related to survival that were not considered in this analysis.

## Competing interests

The authors declare that they have no competing interests

## References

[B1] Ferlay J Shin H Bray F Forman D Mathers C Parkin DM GLOBOCAN 2008, Cancer Incidence and Mortality Worldwide: IARC CancerBase No. 10 [Internet] 2010 Lyon, France: International Agency for Research on Cancer

[B2] Altekruse SF Kosary CL Krapcho M Neyman N Aminou R Waldron W Ruhl J Howlader N Tatalovich Z Cho H SEER Cancer Statistics Review, 1975-2007 2010 Bethesda, MD: National Cancer Institute

[B3] Mutetwa B Taioli E Attong-Rogers A Layne P Roach V Ragin C Prostate cancer characteristics and survival in males of African Ancestry according to place of birth: Data from Brooklyn - New York, Guyana, Tobago and Trinidad Prostate 2010 70 1102 1109 10.1002/pros.21144 20503395 PMC3253621

[B4] Coleman MP Quaresma M Berrino F Lutz JM de Angelis R Capocaccia R Baili P Rachet B Gatta G Hakulinen T Cancer survival in five continents: a worldwide population-based study (CONCORD) Lancet Oncol 2008 9 730 756 10.1016/S1470-2045(08)70179-7 18639491

[B5] Bunker CH Patrick AL Konety BR Dhir R Brufsky AM Vivas CA Becich MJ Trump DL Kuller LH High prevalence of screening-detected prostate cancer among Afro-Caribbeans: the Tobago Prostate Cancer Survey Cancer Epidemiol Biomarkers Prev 2002 11 726 729 12163325

[B6] Data by country: Trinidad and Tobago The World Bank http://data.worldbank.org/country/trinidad-and-tobago

